# Discordance between oncotype DX recurrence score and RSPC for predicting residual risk of recurrence in ER-positive breast cancer

**DOI:** 10.1007/s10549-017-4514-z

**Published:** 2017-11-11

**Authors:** Andrew Dodson, David Okonji, Laura Assersohn, Anne Rigg, Amna Sheri, Nick Turner, Ian Smith, Marina Parton, Mitch Dowsett

**Affiliations:** 1 Ralph Lauren Centre for Breast Cancer Research, The Royal Marsden Hospital NHS Foundation Trust, Fulham Road, London, SW3 6JJ UK; 2 Institute of Cancer Research, 123 Old Brompton Road, London, SW7 3RP UK; 3 Breast Unit, The Royal Marsden Hospital NHS Foundation Trust, Fulham Road, London, SW3 6JJ UK; 4 St George’s University Hospitals NHS Foundation Trust, London, UK; 5 Guy’s and St Thomas’ Hospitals NHS Foundation Trust, London, UK; 6 Royal Free London Hospitals NHS Foundation Trust, London, UK

**Keywords:** Breast cancer, Oncotype DX, Recurrence score, RSPC, prognosis, Recurrence risk

## Abstract

**Purpose:**

Oncotype DX, a gene expression assay widely employed to aid decision making on adjuvant chemotherapy use in patients with primary oestrogen receptor-positive (ER+) breast cancer, produces a recurrence score (RS) related to distant disease recurrence (DR) risk (RS%). In node-negative patients, RS can be integrated with clinicopathological parameters to derive RS-pathology-clinical (RSPC) that improves prognostic accuracy.

**Methods:**

Data were collected on patients having clinically indicated tests with an intermediate clinical risk of distant recurrence, and for whom the decision to prescribe chemotherapy remained unclear. Correlation between RS% and RSPC scores was examined. An agreement table was constructed using risk-categorised data. Association between RS%-derived categorical risk assignments and treatment recommendation was evaluated.

**Results:**

Data on 171 tests (168 patients) were available. Median DR risk by RS% was 11% (range 3–34%), by RSPC it was 15% (range 4–63%). Correlation between RS% and RSPC was 0.702 (*p* < 0.001). RS% classified 57.3% of cases as low-, 32.2% intermediate- and 10.5% high-risk for DR; by RSPC proportions were 33.9, 35.7, and 30.4%, respectively. The number of patients receiving chemotherapy recommendations was: 14/87 (16.1%) categorised as low-risk by RS%, 27/49 (55.1%) as intermediate-risk and 12/13 (92.3%) as high-risk. Of 149 patients recommended for endocrine treatment alone, 28 (18.8%) were categorised by RS% as low-risk but by RSPC as intermediate- or high-risk.

**Conclusions:**

In this group of patients, RSPC assessed fewer patients as low-risk and more as high-risk than did RS%. The discordances between the scores indicate that RSPC estimates of risk should be considered when selecting patients for endocrine therapy alone.

**Electronic supplementary material:**

The online version of this article (doi:10.1007/s10549-017-4514-z) contains supplementary material, which is available to authorised users.

## Introduction

Breast cancer is the most commonly diagnosed invasive cancer in women worldwide. Its incidence rate varies from 19 per 100,000 women in Eastern Africa to 90 per 100,000 women in Western Europe [1]. In the UK, it currently represents 31% of all new female cancers; there were more than 55,000 newly reported cases in 2015. Incidence is rising and in developed countries is predicted to continue to rise by 2% per year for at least the next two decades [2].

In contrast, breast cancer-specific mortality has fallen dramatically over the past 30-years, with the 10-year survival rate for England and Wales currently standing at around 79%, which greatly improves on the figure of 49% for the early-1980s [3]; reduced mortality rates are probably due to a combination of earlier detection and improved treatments. In particular, this improvement can be attributed to the introduction of post-surgical (adjuvant) drug treatments, which have made a huge impact in the treatment of oestrogen receptor-positive (ER+) breast cancer to the extent that more than 85% of women can expect to remain cancer-free 10-years after their initial diagnosis and treatment. However, it is now clear that, while all such women merit endocrine treatment, not all require additional chemotherapy to prevent recurrence of their disease following surgery. Clinical and pathological parameters carry much information about residual risk of distant disease recurrence (DR), and use of these in systematically produced, evidence-based risk indicators such as the nottingham prognostic index (NPI) has a long history in clinical practice [4].

Molecular tests are increasingly being used to provide information about prognosis to aid decision making about the possibility of omitting chemotherapy when low-risk of distant DR is indicated [5]. Oncotype DX Breast Cancer Assay is such a molecular test, commercially provided from genomic health, incorporated (GHI). It produces a recurrence score (RS) for the purpose of prognostication. In the England and Wales, in 2013, it was recommended by the National Institute for Health and Care Excellence (NICE) for use in patients with ER+ , human epidermal receptor-2-negative (HER2−) early advanced breast cancer, assessed on the basis of clinical algorithms to be at intermediate-risk of DR and who would benefit from additional prognostic information to help guide chemotherapy prescribing [6]. It has been funded by NHS England for patients that have node-negative disease since 2015, and are judged by standard clinical and pathological criteria to be at intermediate-risk of distant disease recurrence, and where the decision to prescribe chemotherapy remains unclear.

Tang and colleagues have examined the information provided by RS combined with that derived from traditional clinicopathological features (tumour grade and size and patient age) standardised against endocrine treatment type (tamoxifen or aromatase inhibitor) [7]. Using data derived from node-negative patients entered into the tamoxifen-treatment arm of the National Surgical Adjuvant Breast and Bowel (NSABP) B-14 study [8], and the monotherapy-treatment arms of the translational research cohort in the Arimidex, Tamoxifen Alone or in Combination (TransATAC) study [9], found that the information contained in the two measures were complementary, and that combining them produced an indicator having substantially more prognostic power than either set used singly: they termed this integrated risk estimate RS-pathology-clinical (RSPC). RSPC risk estimates may be derived by means of a web-based tool provided for educational purposes by GHI, which is freely accessible to clinicians [10]. However, few oncologists use RSPC in patient management.

Use of the Oncotype DX RS test is increasing in many countries, and is presently, standard practice in appropriate cases within most NHS clinical breast oncology units in England and Wales. We combined the data from four NHS Breast Units within the London region to compare risk estimates provided by RS and RSPC and to assess the relationship with RS-based clinical recommendations for chemotherapy use.

## Materials and methods

Anonymised data (RS, patient age at surgery, tumour maximum diameter measured at resection, tumour grade, menopausal status, planned endocrine-based treatment type, and final clinical recommendation on addition of chemotherapy) were collected from four London NHS Foundation Trusts (Guy’s and St Thomas’, Royal Free London, The Royal Marsden and St George’s). Data were collected on patients having Oncotype DX tests ordered between January 2015 and September 2016 as part of routine clinical care in-line with NHS England agreed use in patients at intermediate-risk of distant recurrence as assessed using NPI (score > 3.40 and ≤ 5.40) or PREDICT (score ≥ 3% benefit). In the case of The Royal Marsden, this was a consecutive series of all patients tested; for the other three centres, the patient series included only those where there was documented evidence of a clinical recommendation following receipt of the RS result.

The Oncotype DX breast cancer assay reports two measures related to recurrence risk: the RS itself, and an RS-derived percentage estimate of residual 10-year risk of distant DR assuming 5-years’ adjuvant endocrine treatment with tamoxifen, termed RS%. Both RS% and RSPC produce results on a 0–100% scale, and both use the same cut-points to define risk boundaries (vide infra), therefore to maximise comparability, we report here on a comparison between RS% and RSPC.

The published risk categories for RS were originally chosen based on results from the tamoxifen-treatment arm of the NSAPB B-20 trial [8], with scores less than 18 designating low-risk, those between 18 and 30 intermediate-risk, and those above 30 high-risk. On the basis of results from the NSAPB B-14 study, which was used to validate the assay, an RS of 18 equates to a 10-year residual risk of approximately 12% in a tamoxifen-treated population, while an RS of 31 is equivalent to a 21% risk. The same risk cut-points were designated for RSPC by Tang et al. [7] in their paper, and they have been retained here.

RS% was calculated using tamoxifen as the intended endocrine treatment, which is in-line with the default treatment for GHI-reported clinical cases. Tumour size and grade, patient age at surgery and intended endocrine treatment (tamoxifen or aromatase inhibitor) were combined with the RS to produce an RSPC. Web-based tools provided by GHI were used to calculate the RS% and RSPC scores for each study case [10].

The NPI was calculated for each case using the published formula:

$${\text{NPI }} = \, \left[ {0.2 \, x \, S} \right] \, + \, N \, + \, G$$

[where *S* is the size of the index lesion in centimetres, *N* is the node status (0 nodes = 1, 1–4 nodes = 2, > 4 nodes = 3), G is the grade of tumour (Grade I = 1, Grade II = 2, Grade III = 3)] [11].

Correlation between RS% and RSPC scores was assessed using Spearman’s *rho* statistic. RS% and RSPC data were log-transformed prior to analysis. Agreement tables were constructed to compare the agreement of categorical assignments made by RS% and RSPC. RS% and RSPC derived categorical scores were correlated with chemotherapy recommendation.

## Results

### Study cases

A total of 177 cases from 174 patients having tumours tested by the Oncotype DX assay were submitted by the four hospitals’ breast units during the study period (3 patients each had 2 concurrent assays performed on discrete tumours or tumour foci); 6 cases (6 patients) were excluded from analysis, 1 due to node-positive status and 5 due to missing RS result. Centre 3 contributed just under 55% of the cases, Centre 1 approximately 20% and Centres 2 and 4 approximately 12% each. 171/177 cases (96.6%) from 168 patients (96.2%) were analysed for concordance.

An additional of 19 cases (19 patients) were excluded from analysis of treatment recommendation due to unavailable recommendation data; therefore 152 cases (149 patients) were available. Supplementary Table S1 tabulates study and centre-specific case and patient numbers.

Median patient age at surgery was 53 years (range 24–78 years), with pre- and post-menopausal women being almost equally represented (46.6 and 49.4% respectively). Patient demographics showed some centre-to-centre variation; the median age for women in Centre 1’s case-set was 49 years (range 33–74 years), noticeably lower than that for the study overall; at this centre, the proportion of pre-menopausal women was also higher (64.9%). These contrasts with Centre 3, where the median age of its patients was 56 years (range 24–78 years) and 40.4% were pre-menopausal.

Almost all patients were node-negative (94.8%), in-line with NHS England referring guidance for the test. Seven patients (4.0%) presented with one or more micro-metastasis and have been classified as node-negative. The single node-positive patient has been excluded.

Median tumour diameter was 23 mm (range 6–120 mm), with the median diameter for cases from Centres 2, 3 and 4 all being closely similar to this; in contrast, tumours referred for testing at Centre 1 tended to be smaller, with a median tumour diameter of 18 mm (range 6–70 mm).

Tumour grade distribution was closely similar for all centres, with the majority being Grade 2 (58.2%) or Grade 3 (40.1%), only Centre 3 tested any tumours that were Grade 1 (1.1%).

Median and range of NPI was closely similar for all centres. For the whole study, the median was 4.0 (range 2.4–5.4). Similarity in results for this index between centres indicates that although the centre-specific differences existed in the distributions of tumour size and grade, these characteristics were balanced for individual patients.

Patient demographics and tumour characteristics together with information on NPI and treatment recommendations are shown in Table 1.

**Table 1:** Patient demographics and tumour characteristics

	All centres	Centre 1	Centre 2	Centre 3	Centre 4
Gender	*N* patients (%)				
Female	171 (98.3%)	37 (100.0%)	19 (95.0%)	92 (97.9%)	23 (100.0%)
Male	2 (1.1%)	0 (0%)	1 (5.0%)	1 (1.1%)	0 (0%)
Unknown	1 (0.6%)	0 (0%)	0 (0%)	1 (1.1%)	0 (0%)
Age at surgery (years)	Median (range)				
Age at surgery (years)	53 (24–78)	49 (33–74)	51 (37–72)	56 (24–78)	54 (38–74)
Unknown (*N* patients (%))	1 (0.6%)	0 (0%)	0 (0%)	1 (1.1%)	0 (0%)
Menopausal status	*N* patients (%)				
Pre-menopausal	81 (46.6%)	24 (64.9%)	8 (40.0%)	38 (40.4%)	11 (47.8%)
Post-menopausal	86 (49.4%)	13 (35.1%)	10 (50.0%)	51 (54.3%)	12 (52.2%)
Peri-menopausal	2 (1.1%)	0 (0%)	1 (5.0%)	1 (1.1%)	0 (0%)
Not applicable (male)	2 (1.1%)	0 (0%)	1 (5.0%)	1 (1.1%)	0 (0%)
Unknown	3 (1.7%)	0 (0%)	0 (0%)	3 (3.2%)	0 (0%)
Number of involved nodes	*N* patients (%)				
*N* = 0	165 (94.8%)	35 (94.6%)	20 (100.0%)	88 (93.6%)	22 (95.6%)
*N* ≥ 1	1 (0.6%)	0 (0%)	0 (0%)	0 (0%)	1 (4.3%)
Micro-metastasis	7 (4.0%)	2 (5.4%)	0 (0%)	5 (5.3%)	0 (0%)
Unknown	1 (0.6%)	0 (0%)	0 (0%)	1 (1.1%)	0 (0%)
Tumour diameter (mm)	Median (range)				
Tumour diameter	23 (6–120)	18 (6–70)	25.5 (9–42)	25 (10–120)	20 (6–56)
*N* cases (%)					
< 10 mm	6 (3.4%)	3 (8.1%)	1 (5.0%)	0 (0%)	2 (8.7%)
10–19	46 (26.0%)	20 (54.1%)	4 (20.0%)	16 (17.0%)	6 (26.1%)
20–29	62 (35.0%)	9 (24.3%)	8 (40.0%)	34 (36.2%)	11 (47.8%)
30–39	27 (15.3%)	2 (5.4%)	6 (30.0%)	16 (17.0%)	3 (13.0%)
40–49	17 (9.6%)	3 (8.1%)	1 (5.0%)	12 (12.8%)	1 (4.3%)
≥ 50	18 (10.2%)	2 (5.4%)	0 (0%)	15 (16.0%)	1 (4.3%)
Unknown	1 (0.6%)	0 (0%)	0 (0%)	1 (1.1%)	0 (0%)
Tumour grade	Median (range)				
Tumour grade	2 (1–3)	2 (2–3)	2 (2–3)	2 (1–3)	2 (2–3)
N cases (%)					
Grade 1	2 (1.1%)	0 (0%)	0 (0%)	2 (2.1%)	0 (0%)
Grade 2	103 (58.2%)	23 (59.0%)	11 (55.0%)	53 (56.4%)	15 (65.2%)
Grade 3	71 (40.1%)	16 (41.0%)	9 (45.0%)	38 (40.4%)	8 (22.7%)
Unknown	1 (0.6%)	0 (0%)	0 (0%)	1 (1.1%)	0 (0%)
Prognostic indices	Median (range)				
NPI	4.0 (2.4–5.4)	3.8 (3.1–4.9)	3.7 (3.4–4.8)	4.1 (2.4–5.4)	3.7 (3.1–4.7)
RS%	11% (3–34%)	11% (4–34%)	10% (3–30%)	11% (3–34%)	9% (5–23%)
RSPC	15% (4–63%)	14% (4–63%)	14% (5–33%)	18% (5–51%)	13 (6–31%)

Percentages indicate the proportions of patients for age, menopausal status and number of involved nodes; for tumour size and grade percentages they are the proportions of cases. NPI, RS% and RSPC distributions for the whole study and for each centre individually are also given

### RS% and RSPC scores

Median 10-year DR risk estimate by RSPC was 15% (range 4–63%); it was appreciably lower by RS% (11%, range 3–34%) (Table 1). Correlation between RS% and RSPC was statistically significant (*rho* = 0.702, *p* < 0.001, two-tailed), with the *rho* value indicating that just under half of the variation seen in one measure could be explained by variation in the other.

### Agreement analysis for categorical assignment

**Fig. 1:**
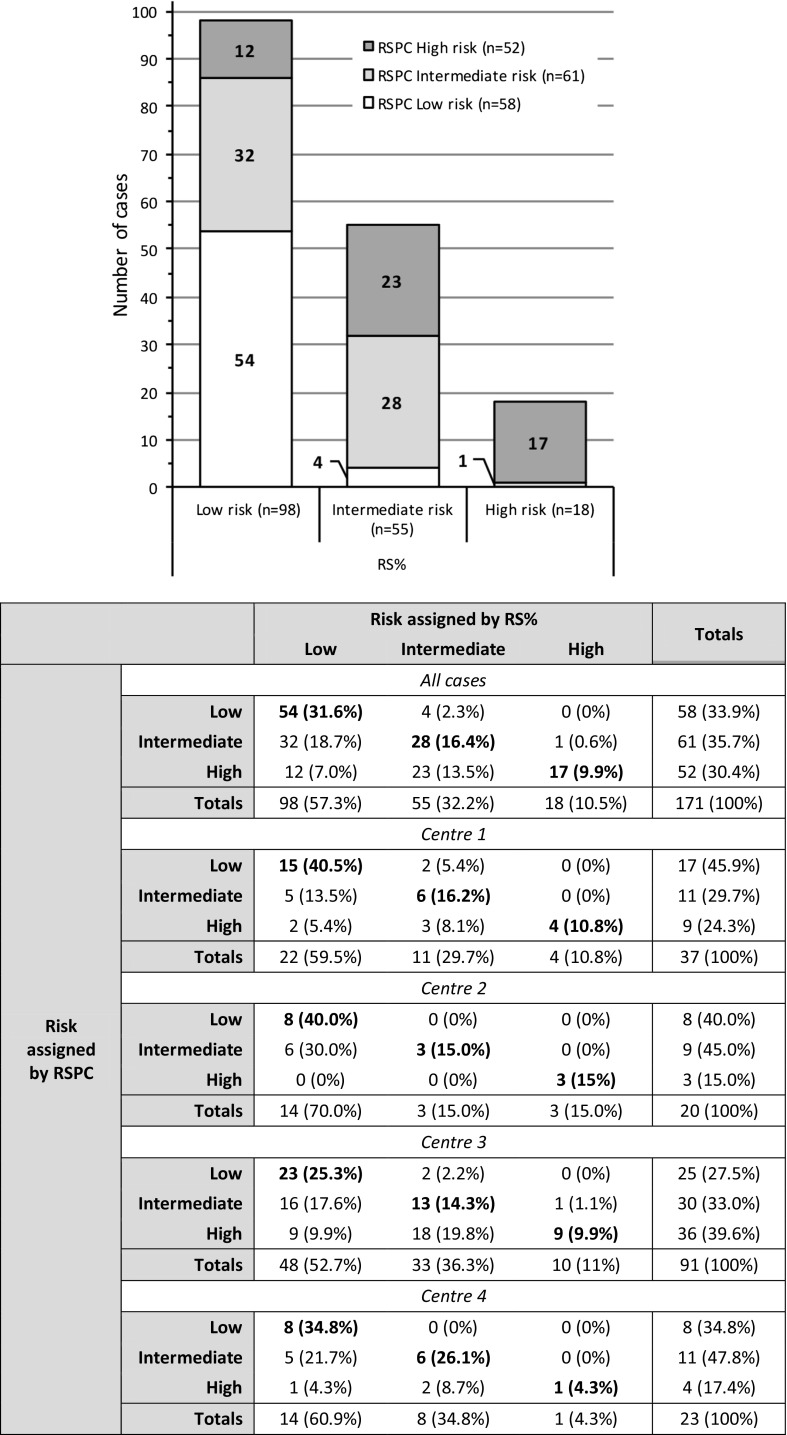
(Combined figure) *Chart*: The histogram shows cross-classification of cases by RS% and RSPC to each risk category. Case numbers were similar for RS% and RSPC with respect to intermediate-risk; however, the number of cases in the low-risk category was very noticeably higher when RS% was used to assign the risk compared to RSPC, and concomitantly, the number designated as high-risk very noticeably lower. *Table*: Contingency table for risk category assignment. Agreement data are shown for all cases in the study and individually for centre-specific cases

Cross-tabulation comparison demonstrated evidence for a considerable non-concordance between RS%- and RSPC-based risk categorisations. The proportions of cases assigned respectively to each of the three risk categories by RS% and RSPC were markedly disparate. When RS% was used to classify cases 57.3% were designated as low-, 32.2% as intermediate-, and 10.5% as high-risk. When RSPC was used, the figures were, 33.9, 35.7 and 30.4%, respectively (see Chart 1 in Fig. 1).

Agreement was seen in a total of 99/171 cases (57.9%); 5 (2.9%) had a lower risk category classification and 67 (39.2%) a higher classification when RSPC rather than RS% was used. Comparing the categorical assignment of cases made by RS% and RSPC in more detail; 32 (18.7%) classified as low-risk by RS% were increased by RSPC to intermediate-risk, and 12 (7.0%) to high-risk; 23 (13.5%) classified as intermediate-risk by RS% were increased to high-risk by RSPC. In contrast, decreased risk-classification of cases by RSPC compared to RS% was seen in only 5 cases, 4 (2.3%) decreased from intermediate-risk to low-risk, and 1 (0.6%) changed from high-risk to intermediate-risk (see Table in Fig. 1).

### Treatment recommendation

Data on treatment recommendations were available on 149/174 patients (85.6%) (Table 2). Overall, within the study, 96 patients (64.4%) were recommended to receive endocrine treatment alone, while there was a recommendation made for adding chemotherapy to endocrine-based treatment for 53 patients (35.6%). There were centre-specific differences seen in the proportions of patients receiving recommendations for the addition of chemotherapy; at Centre 1, the proportion of patients recommended to receive chemotherapy closely matched that of the whole study, while Centres 2 and 4 had similar proportion to each other, both of which were substantially lower than that in the whole study. In contrast, chemotherapy was recommended to a higher proportion of patients whose tests were conducted as part of their care pathway at Centre 3 (for details see Table 2).

**Table 2:** Treatment recommendation according to RS%-designated risk category

Risk assigned using RS%	All patients			Centre 1			Centre 2			Centre 3			Centre 4		
	Endo	Chemo	Totals	Endo	Chemo	Totals	Endo	Chemo	Totals	Endo	Chemo	Totals	Endo	Chemo	Totals
Low	73 (49.0%)	14 (9.4%)	87 (58.4%)	20 (57.1%)	0 (0%)	20 (57.1%)	14 (70.0%)	0 (0.0%)	14 (70.0%)	28 (38.4%)	13 (17.8%)	41 (56.2%)	11 (52.4%)	1 (4.8%)	12 (57.1%)
Intermediate	22 (14.8%)	27 (18.1%)	49 (32.9%)	4 (11.4%)	7 (20.0%)	11 (31.4%)	0 (0.0%)	3 (15.0%)	3 (15.0%)	13 (17.8%)	14 (19.2%)	27 (37%)	5 (23.8%)	3 (14.3%)	8 (38.1%)
High	1 (0.7%)	12 (8.1%)	13 (8.7%)	0 (0%)	4 (11.4%)	4 (11.4%)	1 (5.0%)	2 (10.0%)	3 (15.0%)	0 (0%)	5 (6.8%)	5 (6.8%)	0 (0%)	1 (4.8%)	1 (4.8%)
Totals	96 (64.4%)	53 (35.6%)	149 (100%)	24 (68.6%)	11 (31.4%)	35 (100%)	15 (75.0%)	5 (25.0%)	20 (100%)	41 (56.2%)	32 (43.8%)	73 (100%)	16 (76.2%)	5 (23.8%)	21 (100%)

Data are presented for the whole study and individually for each centre. A total of 149/174 (85.6%) patient’s data were available for analysis (19 patients no treatment recommendation recorded, 6 were excluded for other reasons as detailed in the Results section); centre-specific case availability data are presented in Supplementary Table S1

*Endo* endocrine treatment only, *Chemo* endocrine treatment plus chemotherapy

Stratified by RS%-designated risk category, proportions of patients having a recommendation for the addition of chemotherapy were, 14/87 (16.1%) in the low-risk category, 27/49 (55.1%) in the intermediate-risk category, and 12/13 (92.3%) in the high-risk category. These results clearly indicate an association between RS% result and treatment recommendation, and this association was recapitulated in the individual data from all centres (Table 2).

A substantial number of patients with low-risk indications by RS% but RSPC risk scores indicative of intermediate- or high-risk received recommendations for adjuvant endocrine-based therapy alone. There was a total of 28 such patients (18.8%), 22 (14.8%) with an intermediate-risk and 6 (4.0%) with a high-risk indication by RSPC. Such patients were present in the cohorts submitted by each centre (Table 3).

**Table 3:** Cross-tabulation table for treatment recommendations according to RS% and RSPC designated risk category

	Risk assigned by RS%						Totals	
	Low		Intermediate		High			
	Endo	Chemo	Endo	Chemo	Endo	Chemo	Endo	Chemo
Risk assigned by RSPC								
All patients								
Low	45 (30.2%)	5 (3.4%)	2 (1.3%)	1 (0.7%)	0 (0%)	0 (0%)	47 (31.5%)	6 (4.0%)
Intermediate	**22 (14.8%)**	6 (4.0%)	14 (9.4%)	11 (7.4%)	0 (0%)	1 (0.7%)	36 (24.2%)	18 (12.1%)
High	**6 (4.0%)**	3 (2.0%)	6 (4.0%)	15 (10.1%)	1 (0.7%)	11 (7.4%)	13 (8.7%)	29 (19.5%)
Totals	73 (49.0%)	14 (9.4%)	22 (14.8%)	27 (18.1%)	1 (0.7%)	12 (8.1%)	96 (64.4%)	53 (35.6%)
Centre 1								
Low	13 (37.1%)	0 (0%)	2 (5.7%)	0 (0%)	0 (0%)	0 (0%)	15 (42.9%)	0 (0%)
Intermediate	**5 (14.3%)**	0 (0%)	2 (5.7%)	4 (11.4%)	0 (0%)	0 (0%)	7 (20%)	4 (11.4%)
High	**2 (5.7%)**	0 (0%)	0 (0%)	3 (8.6%)	0 (0%)	4 (11.4%)	2 (5.7%)	7 (20%)
Totals	20 (57.1%)	0 (0%)	4 (11.4%)	7 (20%)	0 (0%)	4 (11.4%)	24 (68.6%)	11 (31.4%)
Centre 2								
Low	8 (40%)	0 (0%)	0 (0%)	0 (0%)	0 (0%)	0 (0%)	8 (40%)	0 (0%)
Intermediate	**6 (30%)**	0 (0%)	0 (0%)	3 (15%)	0 (0%)	0 (0%)	6 (30%)	3 (15%)
High	0 (0%)	0 (0%)	0 (0%)	0 (0%)	1 (5%)	2 (10%)	1 (5%)	2 (10%)
Totals	14 (70%)	0 (0%)	0 (0%)	3 (15%)	1 (5%)	2 (10%)	15 (75%)	5 (25%)
Centre 3								
Low	16 (21.9%)	5 (6.8%)	0 (0%)	1 (1.4%)	0 (0%)	0 (0%)	16 (21.9%)	6 (8.2%)
Intermediate	**9 (12.3%)**	5 (6.8%)	7 (9.6%)	3 (4.1%)	0 (0%)	1 (1.4%)	16 (21.9%)	9 (12.3%)
High	**3 (4.1%)**	3 (4.1%)	6 (8.2%)	10 (13.7%)	0 (0%)	4 (5.5%)	9 (12.3%)	17 (23.3%)
Totals	28 (38.4%)	13 (17.8%)	13 (17.8%)	14 (19.2%)	0 (0%)	5 (6.8%)	41 (56.2%)	32 (43.8%)
Centre 4								
Low	8 (38.1%)	0 (0%)	0 (0%)	0 (0%)	0 (0%)	0 (0%)	8 (38.1%)	0 (0%)
Intermediate	**2 (9.5%)**	1 (4.8%)	5 (23.8%)	1 (4.8%)	0 (0%)	0 (0%)	7 (33.3%)	2 (9.5%)
High	**1 (4.8%)**	0 (0%)	0 (0%)	2 (9.5%)	0 (0%)	1 (4.8%)	1 (4.8%)	3 (14.3%)
Totals	11 (52.4%)	1 (4.8%)	5 (23.8%)	3 (14.3%)	0 (0%)	1 (4.8%)	16 (76.2%)	5 (23.8%)

A total of 149/174 (85.6%) patient’s data were available for analysis (19 patients no treatment recommendation recorded, 6 were excluded for other reasons as detailed in the Results section); centre-specific case availability data are presented in Supplementary Table S1. Figures highlighted in bold indicate cases that might be considered to represent the risk category discrepant cases that have the most potential clinical impact

## Discussion

We report on a comparison between RS%, which is an RS-derived percentage estimate and RSPC, which incorporates clinicopathological parameters. Both produce results on a 0–100% scale, indicative of residual 10-year risk of distant DR assuming 5-years’ adjuvant endocrine treatment with tamoxifen using the same cut-points to define risk boundaries.

**Fig. 2:**
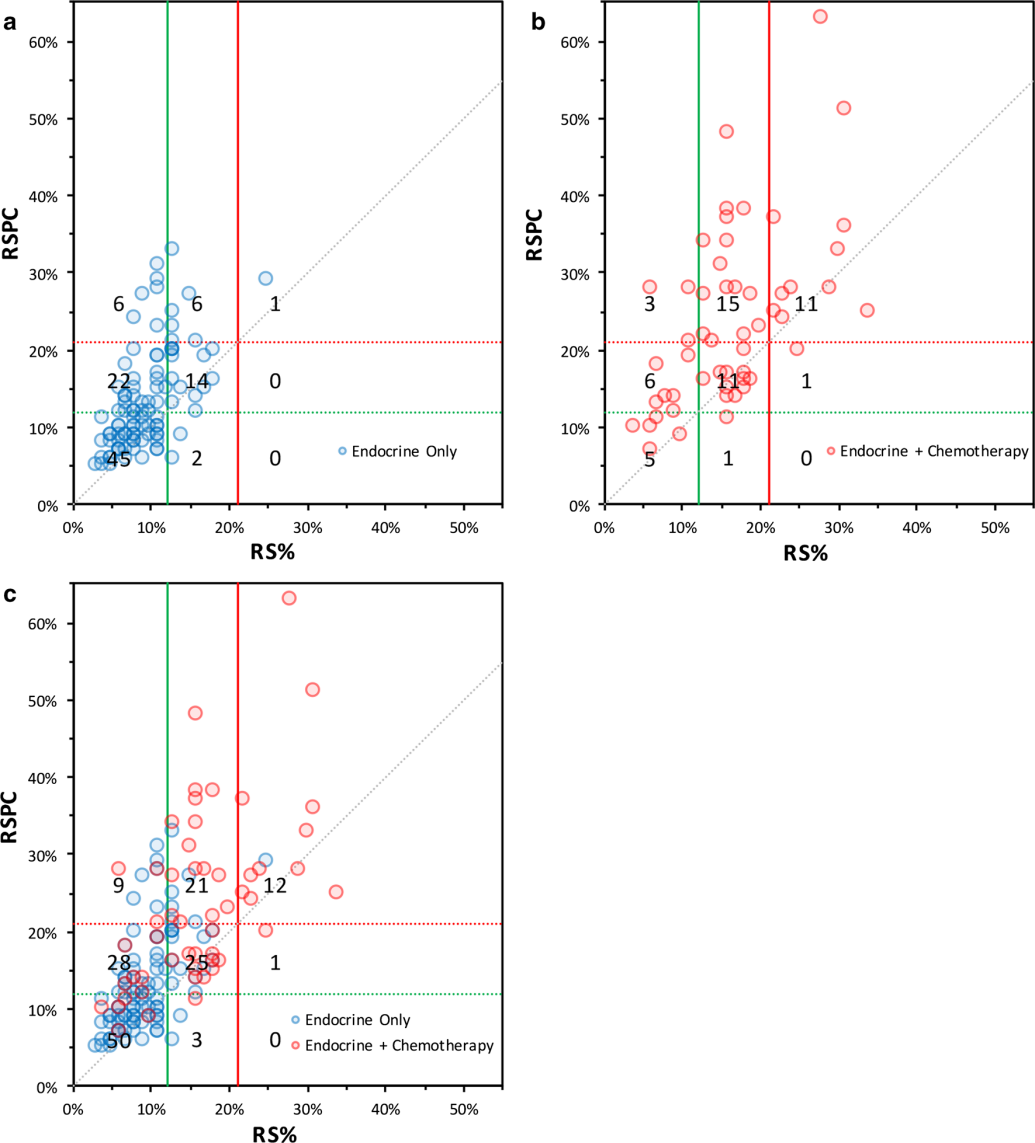
Composite figure comprising three scatterplots showing the position of each patient with regard to their RS% and RSPC scores. **a** shows patients for whom the recommendation was for endocrine treatment alone, indicated by blue circles at the intersection of the RS% and RSPC scores for that patient’s tumour. **b** shows patients in whom the recommendation was for the addition of chemotherapy to endocrine treatment, indicated by red circles. **c** this scatterplot overlays both sets of cases as shown (**a**) and (**b**). In all plots, the green lines indicate boundaries between low-risk and intermediate-risk categories, while the red lines indicate those between intermediate-risk and high-risk; solid lines are applicable to RS% scores, dotted-lines to RSPC scores. The grey dotted line indicates the line of equality. The numbers in each sector are number of patients (note that due to overlying data points, the number of points in the sector may not appear to agree with the figure shown)

In the study by Tang et al., a significant improvement in prognostic performance was seen for RSPC over RS [7]. Similarly, RSPC had substantially improved prognostic performance when compared to clinicopathologic features alone. Here, we show that there is a substantial upward shift in risk categorisation when RSPC rather than RS% score is applied to a contemporary cohort of patients. Considering the effect on treatment recommendations, the most clinically relevant changes are those that cause 37 patients (24.8%) to cross from low- to intermediate-risk, or, low- to high-risk. In this group, 28 (75.7%) received an endocrine only recommendation, which they may not have if the RSPC result had been used in place of RS%. Figure 2(c) shows a scatterplot relating all RS% and RSPC scores. In it, most points clearly lie above the line of equivalence, indicating that even when they do not cross risk boundaries, the indicated risk of DR by is higher by RSPC compared to RS%.

In patients where RSPC increased risk category assignment, median tumour size was greater than for the whole study population (30 vs. 23 mm) and seems to have been the main driver of the increase in risk estimation by RSPC since other demographic and clinicopathological parameters did not differ. Consequently, median NPI was also higher, at 4.3 versus 4.0.

The trend for RSPC results within our series to indicate an increased risk of distant DR as in direct contrast with that seen by Tang et al. in the NSABP B-14 and TransATAC cohorts, where the tendency was RSPC results to classify fewer patients than RS as intermediate-risk (17.8 vs. 26.7%) and more patients as lower risk (63.8 vs. 54.2%). This difference can be explained by the variances in the distributions of patient age, tumour size and most particularly tumour grade that exist in the populations examined by Tang et al. and in our study. The median ages in the B-14, TransATAC and our study were 58, 63 and 53 years, respectively; median tumour sizes were 20, 18 and 23 mm; the proportions of Grade 1 tumours were 35, 22 and 1%, while those for Grade 3 tumours were 20, 18 and 40%. All these measures indicate that the patients within our study represented a population at higher risk, this being an inevitable consequence of eligibility requirements set for the test by NHS England.

Limited evidence has been published indicating that patients with low Oncotype DX scores may gain little from chemotherapy even though their clinicopathological features may indicate they are at high-risk of recurrence [12, 13]. However, central meta-analyses of chemotherapy (anthracycline- or taxane-based) trials in both ER+ and ER- breast cancer patients have produced little or no evidence for stratification of benefit according to clinicopathological features. Rather, they have shown that the benefit from chemotherapy is proportional to overall risk [14]. With the current discordant data, it is unknown if the superior prognostic performance of RSPC compared to RS, it would necessarily translate into improved prediction of benefit from chemotherapy. The data in this current study cannot address the relative predictive value of either score. In particular, it is cannot be known if patients who are converted from low-risk RS to intermediate-risk by RSPC, derive benefit from chemotherapy.

The TAILORx prospective trial examining the use of Oncotype DX scores to stratify treatment in ER+ (and/or progesterone receptor-positive), HER2-, node-negative early advanced breast cancer has reported 5-year results on its low-risk patient cohort assigned to receive endocrine treatment alone [15]. In this group of 1626 patients, the rate of freedom from DR was 99.3% (95% CI 98.7–99.6). Potentially, this is very strong evidence for the effectiveness of the RS alone in assigning risk. However, the eligibility criteria for patients in TAILORx differ very substantially from those in our study; the RS cut-off defining low-risk was ≤ 10 and not ≤ 17, tumours could be up to 50 mm in diameter only if Grade 1, and ≤ 10 mm if Grade 2 or 3. Examination of the data of size distribution for the study shows that the vast majority (92%) were less than 30 mm. In summary, this population’s clinicopathological profile is heavily weighted towards low-risk, such that few (if any) would have a RSPC of > 10%.

Our evidence for the association between RS% and treatment recommendation relies on observed increasing proportions of patients with recommendations for chemotherapy usage in the three ascending RS%-assigned risk categories (16.1, 55.1, 92.3%, respectively). Clinical recommendations of this type do not rely on any single parameter alone, but on a synthesis of multiple observations about tumour biology, clinical presentation, patient co-morbidities and other factors and that the treating clinician and/or the multi-disciplinary team amalgamate to arrive at their final decision regarding treatment recommendations. Countering this as the evidence presented in a recently published retrospective study conducted in the USA taking a look at 431 patients who had their RS result integrated into a multivariable regression analysis together with clinicopathological features. The study found RS indicative of intermediate- or high-risk was the single most influential factor indicating likelihood of chemotherapy use [16].

Currently, Oncotype DX is the only molecular prognostic profiling tool recommended for use in the NHS in England and Wales, but elsewhere the availability of test is broader. For example, American Society of Clinical Oncologists has recently endorsed the use of MammaPrint (agendia NV) for node-negative patients clinically indicated to be at high-risk [17].

There are some limitations to the study presented here and conclusions drawn from its analysis should be viewed with some caution. The sample size is relatively small although the data were largely consistent between the 4 centres despite some differences on the characteristics of recruited patients. We have not been able to present any outcome data; to determine fully the clinical validity of the results, patients would need to be randomised to management according to the 2 scoring methods. All prognostic indices that rely on assessment of clinicopathologic criteria such as grade are potentially subject to confounding intra-observer variability, and this may be a particular problem where strict adherence to standards is not in place. The study’s patient population has been defined using NHS England criteria of node-negative and intermediate-risk by NPI or similar, and this is not universally applicable to tested populations in other parts of the world.

## Conclusion

In a clinically relevant patient population judged by standard clinical and pathological features to be at intermediate-risk of disease recurrence, and in England and Wales as defined by NHS England guidelines for Oncotype DX use, RSPC indicates that substantially fewer patients are at low-risk and substantially more are at high-risk of DR when compared to RS%. Since RSPC produces a superior estimate of risk (but not necessarily of chemosensitivity), it presents a standard option to use in preference to the RS% when decisions about chemotherapy recommendation are being made on the basis of a patient’s risk of DR. The relatively small sample size and absence of outcome data indicate that conclusions should be viewed with some caution and require further validation by additional studies.

## Electronic supplementary material

Below is the link to the electronic supplementary material.

Supplementary material 1 (DOCX 21 kb)
