# Supplementary material for: Discordance between oncotype DX recurrence score and RSPC for predicting residual risk of recurrence in ER-positive breast cancer
Source: Breast Cancer Res Treat. 2017 Nov 11;168(1):249–58. doi: 10.1007/s10549-017-4514-z (PMC5847032; doi:10.1007/s10549-017-4514-z)
Supplement: Supplementary file 1 — Supplementary material 1 (DOCX 21 kb) [file 10549_2017_4514_MOESM1_ESM.docx]

**Discordance between Oncotype DX Recurrence Score and RSPC for predicting residual risk of recurrence of ER-positive breast cancer**

**SUPPLEMENTARY DATA: Table S1.**

Case and patient numbers in the study. Three patients each had two separate tumours or tumour foci concurrently submitted for Oncotype DX testing. Six cases from 6 patients were unsuitable for any further analysis (5 due to missing RS results, and 1 which was a self-funded test in a patient with two tumour-involved axillary lymph nodes). Nineteen further cases from 19 patients were unsuitable for analysis of treatment recommendation due to unavailable information regarding recommendation made.

|  | **All Centres** | **Centre 1** | **Centre 2** | **Centre 3** | **Centre 4** |
| --- | --- | --- | --- | --- | --- |
| *Entered into study* | *N (%)* | | | | |
| cases | 177 (100%) | 39 (22.0%) | 20 (11.3%) | 94 (53.1%) | 24 (13.6%) |
| patients | 174 (100%) | 37 (21.3%) | 20 (11.5%) | 94 (54%) | 23 (13.2%) |
| *Analyses of concordances* | *N (%)* | | | | |
| cases | 171 (100%) | 37 (21.6%) | 20 (11.7%) | 91 (53.2%) | 23 (13.5%) |
| patients | 168 (100%) | 35 (20.8%) | 20 (11.9%) | 91 (54.2%) | 22 (13.1%) |
| *Analyses of treatment recommendation* | *N (%)* | | | | |
| cases | 152 (100%) | 37 (24.3%) | 20 (13.2%) | 73 (48.0%) | 22 (14.5%) |
| patients | 149 (100%) | 35 (23.5%) | 20 (13.4%) | 73 (49.0%) | 21 (14.1%) |

**TABLE S1.**
